# Research progress on endoplasmic reticulum homeostasis in kidney diseases

**DOI:** 10.1038/s41419-023-05905-x

**Published:** 2023-07-27

**Authors:** Dan Wu, Li-Feng Huang, Xiao-Cui Chen, Xiao-Rong Huang, Hui-Yuan Li, Ning AN, Ji-Xin Tang, Hua-Feng Liu, Chen Yang

**Affiliations:** Guangdong Provincial Key Laboratory of Autophagy and Major Chronic Non-communicable Diseases, Key Laboratory of Prevention and Management of Chronic Kidney Disease of Zhanjiang City, Institute of Nephrology, Affiliated Hospital of Guangdong Medical University, 524001 Zhanjiang, Guangdong China

**Keywords:** Podocytes, Acute kidney injury

## Abstract

The endoplasmic reticulum (ER) plays important roles in biosynthetic and metabolic processes, including protein and lipid synthesis, Ca^2+^ homeostasis regulation, and subcellular organelle crosstalk. Dysregulation of ER homeostasis can cause toxic protein accumulation, lipid accumulation, and Ca^2+^ homeostasis disturbance, leading to cell injury and even death. Accumulating evidence indicates that the dysregulation of ER homeostasis promotes the onset and progression of kidney diseases. However, maintaining ER homeostasis through unfolded protein response, ER-associated protein degradation, autophagy or ER-phagy, and crosstalk with other organelles may be potential therapeutic strategies for kidney disorders. In this review, we summarize the recent research progress on the relationship and molecular mechanisms of ER dysfunction in kidney pathologies. In addition, the endogenous protective strategies for ER homeostasis and their potential application for kidney diseases have been discussed.

## Facts

- The endoplasmic reticulum (ER) is an important site for several intracellular biological processes and is essential for maintaining cellular homeostasis.
- Dysregulation of ER homeostasis in renal resident cells triggers the onset and progression of various kidney diseases by promoting renal inflammation and fibrosis.
- Restoration of ER homeostasis through unfolded protein response, ER-associated degradation and autophagy, and ER-overload response presents a therapeutic potential for kidney illnesses.

## Introduction

The endoplasmic reticulum (ER) is the largest organelle in eukaryotic cells, consisting of a series of cavities and thin tubes that interact with each other and form a pipeline system isolated from the cytoplasmic matrix. The ER is a highly dynamic and fluid mesh membrane system [1] and is the main site for intracellular protein synthesis, post-translational modification, folding and transport, oligomerization processing, lipid anabolism, storage and regulation of calcium ions, and signal transduction. Molecular chaperones in the ER, such as glucose-regulated protein 78 (GRP78/Bip), J domain proteins, and lectin chaperones, together with foldase can regulate protein folding and release from the ER. In addition, the ER interacts with other organelles in response to endogenous and exogenous stress [2]. For example, the interaction of ER with mitochondria is beneficial for cellular Ca^2+^ homeostasis [3], whereas its interaction with endosome/Golgi is involved in lipid exchange and metabolism [4]. Moreover, the ER interacts with phagocytes to form autophagosomes and participates in autophagy [5]. Recent studies have shown that various types of stress, including glucose or nutrient deficiency, ischemia and hypoxia, dysregulation of redox state, viral infections, drugs, toxins, and increased synthesis of secreted proteins, may disrupt ER homeostasis [6, 7]. During ER stress, the ability of the ER to process or transport proteins and regulate Ca^2+^ release and uptake is impaired, which causes the accumulation of unfolded and misfolded proteins in the lumen of the ER and an imbalance of calcium homeostasis and lipid synthesis. However, several protective mechanisms, including unfolded protein response (UPR), ER-associated degradation (ERAD), and ER-related autophagy or ER-phagy, are activated during ER stress to restore homeostasis [8].

Increasing evidence has proven that dysregulation of ER homeostasis acts as a key player in the progression of various kidney diseases and may represent a potential therapeutic target. In this review, we focused on research articles with experimental data, which remarkably promote our understanding of the functional mechanisms of ER in kidney disorders, published in the past 10 years. The publications were searched on the PubMed database. “Endoplasmic reticulum” and “kidney disease” were set as search keywords, and original articles and a few reviews in English were collected. We reviewed a small number of studies on the application of drugs targeting ER in kidney illnesses; several studies were not considered to avoid duplication and because of space constraints.

## Maintenance mechanisms of ER homeostasis

### Unfolded protein response (UPR)

Under adverse conditions, such as starvation, hypoxia, calcium imbalance, increased biosynthetic demand, or drug influence, ER folding capacity is often impaired, resulting in the dysregulation of ER homeostasis and the accumulation of a large number of unfolded or misfolded proteins in the ER. Therefore, any disturbance in the ER environment that impairs ER folding ability can trigger ER stress. However, UPR is activated in response to ER stress to normalize ER function. It has recently been suggested that toxic lipid stimuli, such as high levels of saturated fatty acids (FAs), can trigger ER stress by directly acting on membrane fluidity regardless of the level of folded ER proteins [9]. UPR is typically triggered by three canonical UPR mediators (sensors), including inositol-requiring enzyme 1α (IRE1α), protein kinase R-like ER kinase (PERK), and activating transcription factor 6 (ATF6) pathways. These mediators bind to the ER chaperone 78-kDa glucose-regulated protein (GRP78), also referred to as binding immunoglobulin protein (BiP), in a monomeric and inactive form through their luminal domain. Under stress, GRP78 dissociates from the ER and assists in the folding of nascent proteins, activating PERK and ATF6 sensors and their corresponding downstream signaling pathways [10]. However, the three sensors are activated under lipotoxic stress, regardless of the ER protein load [9].

IRE1α is the most evolutionarily conserved ER stress sensor. IRE1α has a ribonuclease domain and can initiate unconventional mRNA splicing of X-box-binding protein 1 to generate an active transcription factor, the spliced form of X-box binding protein 1 (XBP1s). XBP1s in turn enters the nucleus, upregulates the expression of molecular chaperones, folding enzymes, and ER-related degradation factors in the lumen of the ER, and enhances the ability of the ER to process and clear unfolded proteins. IRE1α also functions as an endoribonuclease that degrades several mRNAs through the regulated IRE1α-dependent decay (RIDD) pathway. RIDD is an important component of UPR and has a protective effect on cells [11, 12]. Recently, studies have reported about the regulation of IRE1α activity. For example, the HSP47 protein, a member of the heat shock protein family, has been found to act as a regulator of IRE1α. Under ER stress, IRE1α separates from GRP78, and at that point, HSP47 can competitively bind to IRE1α and promote phosphorylation activation of IRE1α. As HSP47 occupies the theIRE1α-binding site, GRP78 may have only limited ability to return to interact with IRE1α, thus HSP47 could serve as a novel IRE1α regulator. Furthermore, the ER co-partner DnaJ family protein ERDJ4, a cofactor of GRP78, can indirectly regulate the activation of IRE1α, as the affinity of GRP78 to IRE1α is regulated by conformational changes that are dependent on the levels of adenosine triphosphate (ATP). When ATP is hydrolyzed, GRP78 forms a closed conformation and can bind to IRE1α stably. However, the intrinsic ATPase activity of GRP78 is weak, and j-protein cochaperones, such as ERDJ4, can accelerate ATP hydrolysis, improving substrate recognition and GRP78-binding efficiency [8]. Interestingly, activation of IRE1α is also involved in ER-mediated apoptosis. IRE1α-recruited tumor necrosis factor receptor correlation factor 2 activates apoptotic Jun N-terminal kinase or caspase-12 signaling, leading to apoptosis. In addition, RIDD can promote apoptosis, mainly through the degradation of GRP78 mRNA, reducing the expression of molecular chaperones [11, 12].

As an ER-type transmembrane protein belonging to the cyclic adenosine monophosphate (cAMP)-response element binding protein (CREB) transcription factor family, ATF6 can exist in two configurations, ATF6α and ATF6β. The N-terminal cytoplasmic region of ATF6 has a basic region, the leucine zipper (bZIP) DNA transcription activation domain. Free ATF6 is transported into the Golgi in the form of vesicles with the help of coat protein II (COPII) and cleaved by site 1 protease (S1P) and S2P on the Golgi membrane to release an intracellular fragment p50-ATF6, which is translocated into the nucleus and combined with the universal nuclear transcription factor Y (NF-Y) fragment to form a heterodimer. The complex recognizes the specific sequence of the ER stress response element (ERSE) and upregulates the expression of molecular chaperones, such as GRP78, GRP94, and calreticulin, to enhance the protein folding ability of ER. In addition, the complex can promote the expression of XBP1, C/EBP homologous protein (CHOP), and other factors, and work together with transcription activation factor 4 (ATF4) to activate the ER-associated degradation (ERAD) pathway to alleviate ER stress [13]. Currently, it is considered that the ATF6 signaling pathway is mainly involved in promoting cell survival [14].

PERK is a serine/threonine protein kinase that is activated by autophosphorylation under ER stress and acquires full catalytic activity to further phosphorylate eukaryotic translation initiation factor 2α (eIF2α), inhibiting protein translation, and reducing the entry of new proteins into the ER. The PERK-eIF2α pathway activates ATF4 and selectively induces the expression of UPR target proteins, including chaperone and oxidative detoxification enzymes (glutathione-S-transferase and heme oxygenase-1) to reduce cellular oxidative damage and ER stress. Moreover, PERK can increase the transcription levels of CHOP by upregulating ATF4. CHOP can upregulate growth arrest and DNA damage-inducible protein 34 (GADD34) levels. The GADD34 protein can play a negative feedback role in the dephosphorylation of eIF2 to restore normal cell function [15].

The three signaling pathways are not completely independent but interact with each other to share some common proteins. The three-pronged axis orchestrates the UPR process, and their regulatory interdependence is well documented. For example, downstream target genes of ATF6 can be compensated by XBP1 during acute silencing of ATF6. Inhibition of PERK could lead to compensatory activation of XBP1s, whereas the inhibition of IRE1α contributes to the continuous activation of PERK and CHOP [16]. The coordinated effects of misfolded protein degradation and chaperone-assisted protein folding can alleviate ER stress and reestablish ER homeostasis. However, the activation of the maladaptive branch in the ER stress response can induce unresolved chronic ER stress. Under this chronic ER stress condition, the UPR transforms from a pro-survival signal into a pro-apoptotic signal and initiates apoptosis by activating signaling molecules, including CHOP, caspase-12, c-Jun N-terminal kinase (JNK), and B-cell lymphoma 2 (Bcl-2)-associated X protein (Bax) [6].

### ERAD and autophagy

Apart from UPR, which prevents new protein synthesis and promotes the correct folding of existing proteins, ERAD is another key quality control mechanism of ER homeostasis that is responsible for clearing misfolded proteins from the ER via cytoplasmic proteasomal degradation [8]. Peptides that stick to the ER membrane are cleared by the membrane zinc metalloprotease (ZMPSTE24). Some of the polypeptides that successfully enter the ER but are not properly folded may be refolded in the ER, whereas the other misfolded proteins will be cleared. As the ER does not contain a degradation mechanism, the misfolded proteins are transported to the proteasome for degradation after ubiquitination through a process known as the ERAD pathway. ERAD is initiated by the recruitment of unfolded substrates, aided by chaperones (such as the GRP78) and proteins belonging to ER degradation-enhancing α-mannosidase-like protein (EDEM) family. Studies have shown that the inhibition of ERAD can lead to several organ dysfunctions in mouse models, including enteritis, obesity, and glucose intolerance, making it a potential therapeutic target for treating some diseases, including cancer [16]. However, ER-to-lysosome-related degradations, including macroautophagy (also known as autophagy) and ER-specific autophagy (ER-phagy), are necessary for the clearance of abnormally aggregated proteins that cannot be recognized by ERAD partners or that are too large to be re-transported to the cytoplasm [17].

Autophagy is a physiological process tightly regulated by several molecules and autophagy-related genes (ATGs) that are involved in maintaining cellular homeostasis. Autophagy involves two key pathways, the Atg12-Atg5-Atg16 and Atg8/microtubule-associated protein 1 light chain 3 (LC3) pathways. LC3/Atg8 covalently binds to phosphatidylethanolamine (PE), and the soluble form of LC3 (LC3-I) is converted to LC3-II after binding to PE, which is a classic marker for autophagy. Autophagy not only reduces ER load by degrading abnormally aggregated proteins, such as misfolded or unfolded proteins, but also releases degraded products and provides materials for the synthesis of other new proteins, indicating that autophagy contributes to the maintenance of ER homeostasis [10]. Margariti et al. demonstrated that the IRE1/XBP1s and IRE1/JNK axis activate the autophagy inducer beclin-1 [18]. Chen et al. showed that the induction of the PERK/eIF2α/ATF4 axis is critical for ATG expression [16]. However, unlike ERAD, which is limited to protein degradation, ER-related autophagy can be divided into ER stress-mediated autophagy and ER-phagy. The former is involved in the degradation of damaged proteins and organelles, whereas the latter selectively degrades parts of the ER through ER-phagy receptors [12]. ER stress-induced autophagy has two main functions. The first is the formation of ER-containing autophagosomes (ERA), which engulf ER or aggregated proteins that cannot be processed by other pathways. The second function is the reduction of the expanded ER level to normal levels after the alleviation of ER stress. Unlike general autophagy, ER-phagy occurs continuously under normal conditions and is enhanced during starvation. Recent research has identified eight ER-phagy receptors in mammals, including family with sequence similarity 134, member B (FAM134B), SEC62, reticulon-3L (RTN3L), cell-cycle progression gene 1 (CCPG1), atlastin GTPase 3 (ATL3), testis-expressed protein 264 (TEX264), tripartite motif containing 13 (TRIM13), and Calcium Binding And Coiled-Coil Domain 1 (CALCOCO1) [19]. However, little is known about the regulatory mechanisms of ER-phagy.

### ER-overload response (EOR)

In contrast to UPR, EOR initiates other survival-promoting mechanisms to counteract the signal regulation of ER stress induced by prolonged storage of properly folded protein in the ER. This process involves a relatively independent signal path, the EOR-Ca^2+^-ROS-NF-κB (nuclear factor kappa B) pathway. The main mechanism involves the activation of NF-κB to initiate the expression of multiple pro-inflammatory proteins and cell adhesion molecules and regulate cell apoptosis [6]. However, further research is needed to elucidate the relevant mechanisms.

## Dysregulation of ER homeostasis in kidney diseases

Recently, it was estimated that more than 850 million people worldwide are affected by kidney disorders, and approximately 10% of adults are affected by some form of chronic kidney diseases (CKD) [20]. The global burden of acute kidney injury (AKI)-related mortality rates currently far exceeds that of breast cancer, heart failure, or diabetes [21]. Diabetic nephropathy (DN), which affects approximately one in three people with diabetes, is listed as the leading cause of end-stage kidney disease worldwide [22]. CKD is expected to be the fifth leading cause of death globally by 2040 [23].

### ER stress in CKD

**Fig. 1:**
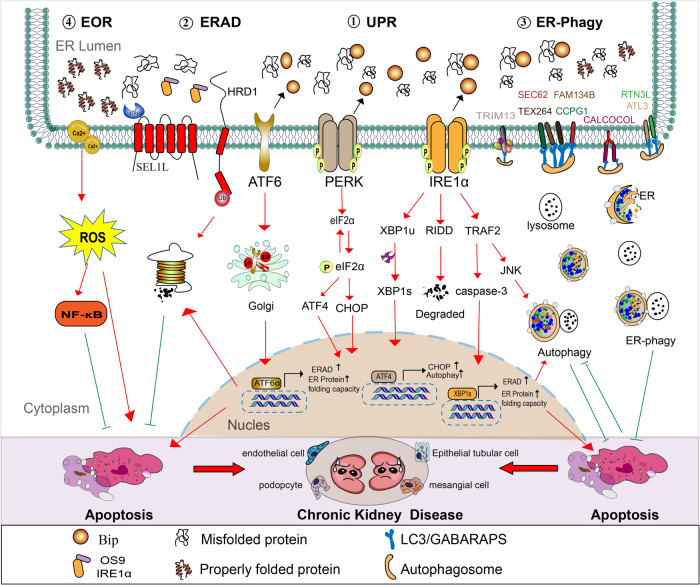
Restoration of ER homeostasis presents a therapeutic potential for the treatment of chronic kidney diseases. In chronic kidney diseases, various pathogenic factors, including free fatty acid, angiotensin II, advanced glycation end products, and hyperglycemia, disrupt ER homeostasis characterized by the accumulation of massive misfolded proteins. UPR, ERAD, autophagy or ER-phagy, and EOR were induced by ER stress to restore ER homeostasis. However, persistent activation of UPR, comprised of at least three UPR stress sensors IRE1 α, PERK, and ATF6, triggers apoptosis in renal intrinsic cells, resulting in the progression of kidney diseases. Eight mammalian ER-phagy receptors have been identified, including FAM134B, SEC62, RTN3L, CCPG1, ATL3, TEX264, TRIM13, and CALCOCO1.

Previous studies have shown that ER function is important for protein homeostasis in the kidneys, and ER stress is involved in primary glomerulonephritis and secondary glomerular disease. Studies have reported an increase in the levels of ER stress markers in renal biopsy samples in patients with minimal change disease, focal segmental glomerulosclerosis, membrane nephropathy, and proliferative glomerulonephritis. ER stress is an important factor in the decline in kidney function towards CKD and end-stage renal disease (ESRD) in patients with diabetes and/or hypertension [24]. Some studies have confirmed that chronic ischemia-induced proteinuria and ER stress can promote tissue remodeling and CKD progression [25, 26]. Mohammed-Ali et al. observed an increase in the expression of ER stress key genes (Grp78, Chop, Atf6, and pleckstrin homology-like domain family A member) and the simultaneous occurrence of albuminuria and renal lesions with UPR activation during the early stages of CKD, confirming that UPR is involved in CKD [27]. In inherited single-gene kidney disorder, mutations in collagen nephropathy collagen type IV alpha 5 chain (COL4A5) and COL4A3 can cause upregulation of ER stress markers (GRP78, calnexin, calreticulin, GADD34, XBP1s, and CHOP) and ERAD markers (ER degradation enhancement α-mannosidase-like protein, EDEM protein) [28, 29]. Moreover, uromodulin (UMOD) mutations result in its retention within the ER of renal tubular cells [30], confirming that ER stress plays an important role in the development of kidney injury. In addition, it is more intuitive that dilated and enlarged ER lesions have been observed in biopsy samples from patients with membranous nephropathy using an electron microscope. ER retention of misfolded proteins in the mesangial and tubular epithelium has been observed in mesangial proliferative glomerulonephritis and DN [24]. Mice with GRP78 mutations may develop severe tubulointerstitial lesions with age [31]. Moreover, podocyte IRE1α-deficient mice have been shown to exhibit podocyte damage, including dilated ER and mitochondria damage [32]. SEC63 and XBP1 double-defective mice reportedly exhibited podocyte apoptosis; in contrast, the intact XBP1 pathway can alleviate stress in the ER and maintain normal glomerular filtration barrier [33]. A recent study showed that simultaneous inactivation of XBP1 and SEC63 in the collecting duct also induces inflammation and activation of myofibroblasts, leading to chronic tubulointerstitial kidney injury [34, 35]. In addition, in animal models of polycystic kidney disease, the accompanying inactivation of SEC63 and XBP1 in distal renal tubules significantly reduced the maturation of polycystic protein-1 and aggravated the polycystic kidney phenotype [36] (Fig. 1).

### ER stress in AKI

Drug-induced nephrotoxic AKI, including cisplatin-induced AKI, is characterized by ER stress [37, 38]. The activation of UPR during AKI can prevent the negative effects of ER stress in the short term [38, 39]. However, persistent activation of UPR can also worsen AKI. For example, Wang et al. reported that intermedin (IMD), a new member of the family of calcitonin/calcitonin gene-related peptides, can prevent renal ischemia–reperfusion injury (IR) by inhibiting ER stress-induced apoptosis [40]. Generally, it is believed that mild to moderate ER stress in AKI promotes cell survival and plays a cytoprotective effect, whereas severe ER stress accelerates apoptosis, indicating the concept of ER stress as a “double-edged sword”. Further studies are necessary to extensively elucidate the mechanism of ER stress in AKI. Research findings suggest that ER stress activation can lead to inadequate renal remodeling during the transition from AKI to CKD. ER stress has been shown to alter the characteristics of renal tubular epithelial cells, promote epithelial-to-mesenchymal transition, induce cell reprogramming, and promote fibrosis, leading to the loss of normal kidney structure [22, 31]. Chronic persistent inflammation is a driver of uncontrolled healing and tissue damage in AKI-to-CKD.

Recent studies have shown that persistent ER stress can act as a driver of inflammatory signaling, exacerbating the activation of major UPR branches (IRE1α/XBP1, PERK/ATF4, and ATF6), inducing the expression of numerous genes involved in inflammation, cell death, autophagy, and oxidative stress. For example, sustained activation of the UPR pathway (IRE1α) can lead to the activation of tumor necrosis factor receptors and the transcription factor AP-1, which in turn promotes the activation of pro-inflammatory pathways, such as NF-κB, nucleotide-binding oligomerization domain (NOD) proteins NOD1/2, and receptor-interacting protein kinase (RIP)-dependent cascades. PERK activation and eIF2α phosphorylation increase the stability of NF-κB. ER stress in macrophages has been shown to promote NF-κB-driven pro-inflammatory phenotypic differentiation, manifested by an increase in pro-inflammatory cytokines, such as interleukins IL-1β and IL-18 [22, 41]. In summary, ER stress and UPR may cause fibrosis by inducing apoptosis, myofibroblast differentiation, epithelial–mesenchymal transition, pro-inflammatory macrophage polarization, and Ca^2+^ release into the cytoplasm matrix, leading to various pathological changes [42, 43].

**Fig. 2:**
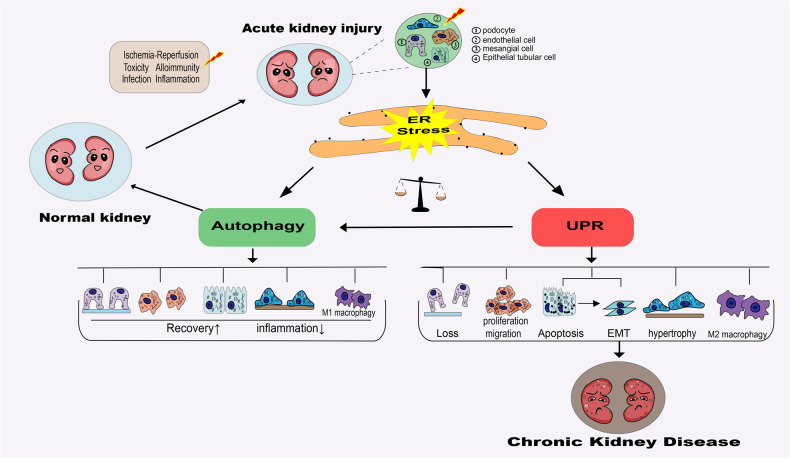
Role of ER stress in AKI-CKD. Various pathogenic factors, such as ischemia, toxicity, infection, and inflammation, cause ER stress in renal intrinsic cells. Autophagy induced by ER stress promotes recovery and restricts renal inflammation. In contrast, persistent activation of UPR impairs recovery progress. Severe ER stress leads to maladaptive repair in the transition from AKI to CKD.

Notably, ER stress is critical in AKI-to-CKD progression [44]. Jao et al. found that ATF6α can disrupt fatty acid metabolism in the proximal renal tubule in ischemia/reperfusion injury (IRI), leading to lipotoxicity-mediated apoptosis and upregulation of connective tissue growth factor (CTGF) and tubulointerstitial fibrosis [45]. Moreover, the production of reactive oxygen species (ROS) during renal IRI is also involved in the pathogenesis and progression of CKD. Nuclear factor E2-related factor 2 (Nrf2) acts as an antioxidant transcriptional regulator that can resist oxidative stress by activating antioxidant genes, such as catalase, heme oxygenase-1 (HO-1), and superoxide dismutase. However, studies have shown that Nrf2 is a downstream target for the ATF6, IRE1/JNK, and PERK pathways [14, 46, 47]. Genetic studies of the reticulon-1 (RTN1) protein, an ER-forming protein primarily localized in the ER, have also confirmed the role of ER stress in AKI-to-CKD progression. In an obstruction-induced AKI model, inhibition of *RTN1* expression attenuated ER stress, apoptosis, and renal fibrosis [48]. Moreover, Fan et al. found that multiple markers of ER stress, including RTN1A, were expressed in kidney biopsy samples in patients with AKI, and their expression levels were positively correlated with AKI severity [49]. Furthermore, studies have shown that ER stress or ER stress-dependent UPR activation also regulates the expression of vascular endothelial growth factor [50], which also plays an important role in the maintenance and survival of endothelial cells during AKI. Fibroblast growth factor 1 (FGF1) therapy can inhibit diabetes-induced ER stress, and FGF10 can attenuate renal IRI-induced kidney cell apoptosis in AKI by reducing the UPR [51, 52]. In addition, Zhang et al. showed that erlotinib-induced inhibition of the estimated glomerular filtration rate (eGFR) alleviated the development of diabetic nephropathy in type 1 diabetes, partially mediated by inhibition of the mechanistic target of rapamycin (mTOR) and activation of AMP-activated protein kinase (AMPK), and is accompanied by increased level of autophagy and inhibition of ER stress [53]. Moreover, Thitinun et al. found that the production of renal erythropoietin (EPO) is significantly related to ER stress, especially the activation of transcription factor ATF4, which can inhibit the 3' enhancer activity of *EPO* [54]. The specific mechanisms of ER stress-driven AKI or AKI-to-CKD progression are not well elucidated (Fig. 2).

### ER-associated autophagy and ER-phagy in kidney diseases

As a cellular homeostatic mechanism, autophagy plays a key role in several cellular physiological and pathophysiological conditions, including cell growth, differentiation, and death, and the regulation of energy balance. In the basal state, cell autophagy is usually low, mainly for the degradation of long-lived proteins and organelles. It has been suggested that at least a part of the membrane of autophagosomes originates from the ER membrane [55]. Evidence suggests that autophagy is associated with UPR and is necessary (other than ERAD) for the clearance of misfolded proteins from secretory pathways. Although ER stress and autophagy can function independently, they possess some common features, including protecting cells by alleviating stress and inducing cell death under extreme stress conditions [24]. ER stress induces autophagy as a mechanism to protect cells from apoptosis [56]. The PERK pathway plays a crucial role in triggering autophagy, and downstream ATF4 and CHOP have been shown to transcribe multiple ATGs, such as *LC3B*, *ATG5*, *ATG12*, *beclin1*, and *ATG16L1*. Inhibiting the expression of PERK can reduce autophagy. Autophagy can balance ER expansion induced by ER stress, improve cell survival rate or induce non-apoptotic death according to the environment [57]. Kawakami et al. reported that treatment with the classic ER stress inducer tunicamycin resulted in a significant increase in LC3-II expression(the marker of autophagy) in the proximal renal tubular cells of the kidneys, indicating that ER stress activates autophagy [58]. Autophagy is an adaptive mechanism for cell survival during ER stress, and inhibiting autophagy can accelerate cell death. Interestingly, in 2015, Dong et al. found that autophagy is activated under low ER stress to overcome mTOR inhibition and prevent apoptosis to promote cell survival. Conversely, autophagy may be blocked in response to severe ER stress to inhibit apoptosis via mTOR activation [59]. Recently, Dong et al. further investigated the upstream state of ER stress in the study of chronic fibrosis animal model induced by tunicamycin. PERK-eIF2α pathway activates autophagy, which may antagonize ER stress and provide a negative feedback mechanism to alleviate cell stress. These findings indicate the crosstalk between ER stress and autophagy in chronic kidney injury and fibrosis [42].

Eight ER-phagy receptors have been discovered [19], but their roles in kidney pathologies have not been identified. Recently, Jiang et al. found that quantum dot-induced nephrotoxicity models not only disrupted ER ultrastructure but also induced UPR and FAM134B-dependent ER-phagy [60]. Huang et al. argued that TRIM13, which reduces renal cell carcinoma metastasis and invasion, could serve as a candidate prognostic marker and potential therapeutic target for renal cell carcinoma [61]. In addition, Li et al. reported that TRIM13 could serve as a potential target for the treatment of diabetic nephropathy [62]. Analysis of the Woroniecka Diabetes Study dataset showed that the ER-phagosome pathway and interleukin–interferon signaling are overactivated and extracellular matrix (ECM) components are overexpressed in the kidneys of patients with diabetes [63]. In the future, analyzing the mechanism of ER-associated autophagy and ER-phagy will improve the understanding of the pathogenesis of kidney illnesses and become a new therapeutic target.

## ER crosstalk with other organelles in kidney diseases

### ER crosstalk with mitochondria

Direct interaction of membrane contact sites (MCS) in organelle has been receiving increasing attention [64]. Contact between ER and mitochondria occurs most often, and the part of the ER that is directly connected to the mitochondria is called the mitochondria-associated ER membrane (MAM) [65]. MAMs are considered the signal hubs for lipid and Ca^2+^ transfer between mitochondria and ER. MAM plays an important role in Ca^2+^ signaling, lipid homeostasis, mitochondrial dynamics, ER stress, apoptosis, inflammation, and autophagy [66, 67]. ER requires high levels of Ca^2+^ in the ER lumen to function properly, and changes in ER Ca^2+^ homeostasis can lead to rapid accumulation of misfolded proteins, activating the UPR. Mitochondria and ER contact at the MAM regulates Ca^2+^ signaling and activates ATP production to meet energy demands and accelerate the removal of misfolded proteins in the ER. However, excessive and persistent increase in Ca^2+^ level can open mitochondrial permeability transition wells (mPTP) and release cytochrome *c*, leading to apoptosis [68]. Studies have shown that MAM integrity is strongly associated with the progression of kidney disorder. Igwebuike et al. confirmed that MAM integrity disruption occurs in the early stages of gentamicin-induced AKI and precedes downstream UPR activation and cell death [69]. Yang et al. found that MAM integrity was impaired in renal biopsy samples of patients with DN, and the kidneys of streptozotocin (STZ)-induced diabetic mice, which was inversely correlated with lipid levels and kidney injury. In addition, the expression of MAM control proteins (disulfide-bond A oxidoreductase-like protein (DsbA-L), phosphofurin acidic cluster sorting protein 2 (PACS-2), and mitofusin 2 (MFN-2) was modulated at different stages of DN [65, 70, 71]. DsbA-L acts as an antioxidant to reduce ER stress, and the reduction in DsbA-L expression disrupts MAM integrity. In contrast, DsbA-L overexpression can inhibit apoptosis by maintaining MAM integrity and MFN-2 expression and improve renal damage [70]. PACS-2 deficiency not only disrupts MAM integrity but also prevents mitochondria formation and mitochondrial autophagy in the proximal renal tubules under diabetic condition [72, 73]. MFN2 mediates mitochondrial dysfunction by activating the PERK pathway, leading to a decrease in MAM levels and apoptosis of podocytes [74]. Overexpression of MFN2 improves Cu-induced MAM dysfunction and increases autophagy [75].

Moreover, RTN1A overexpression can worsen ER stress and mitochondrial dysfunction of renal tubular epithelial cells under diabetic conditions by regulating ER–mitochondrial contact [76]. Excessive vanadium exposure can induce ER–mitochondrial dysfunction, whereas inhibiting inositol triphosphate receptors (IP3R) improved ER mitochondrial dysfunction and attenuated vanadium-induced apoptosis in duck tubular epithelial cells [77].

**Fig. 3:**
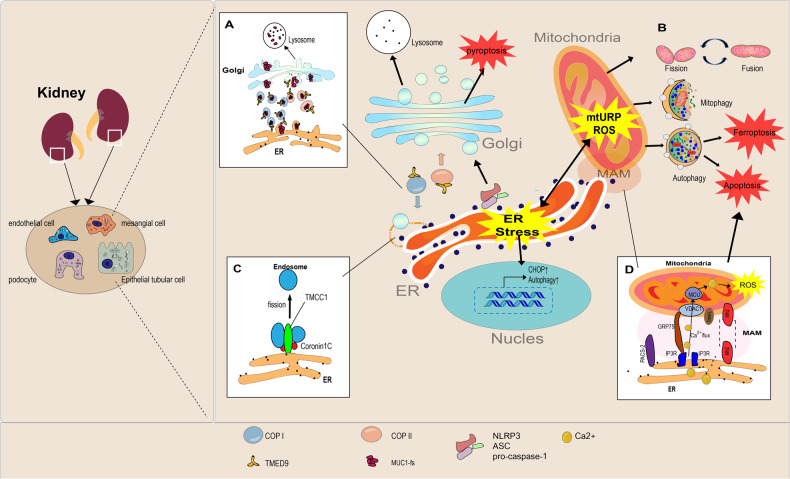
ER interact closely with other organelles to maintain the function of kidney intrinsic cells. **A** Bidirectional membrane trafficking between ER and Golgi is mediated by COPI and COPII. When the MUC1 fs protein is trapped in the vesicles containing TMED9 cargo receptors in the early secretion pathway, and cannot promptly be degraded by the lysosome, the accumulation of toxic MUC1 leads to mucin 1 nephropathy (MKD). **B** ER stress induces ROS production in mitochondria. The interface between the Golgi apparatus, ER, and mitochondria is an important hub for the activation of NLRP3 Inflammasome to cause pyroptosis. **C** Transmembrane and coil domain family 1 (TMCC1) concentrates at the ER-endosome membrane contact sites and controls ER-associated bud fission and subsequent cargo sorting to the Golgi. **D** The part of ER directly connected with mitochondria is called mitochondrial associated ER membrane (MAM), which is composed of a variety of proteins, including inositol triphosphate receptor (IP3R), voltage-dependent anion channel (VDAC), glucose-regulated protein 75 (GRP75), and fibroin 2 (Mfn2) PACS-2, DsbA-L. Calcium can be transported from ER to mitochondria. Mitochondrial fission also occurs at the MAM site. In addition, MAM is closely related to autophagy, mitophagy, and ferroptosis.

In addition, crosstalk between the two organelles can be regulated through the UPR signaling pathway. Studies have shown that under pathogenic conditions of unilateral IRI-induced tubulointerstitial fibrosis, ATF6-induced decrease in peroxisome proliferator-activated receptor α (PPARα) expression downregulates the expression of downstream genes of mitochondrial β oxidation, leading to lipid accumulation and tubular fibrosis [78]. There was a decrease in ER stress and mitochondrial fragmentation in a unilateral ureteral obstruction (UUO) model of CHOP-deficient mice (with no expression of PERK, IRE1α, and ATF6). CHOP acts as a regulator during mitochondrial fission, upregulating the expression of fission and fusion mitochondrial proteins such as Fis1 and Opa1, promoting a reduction in mitochondrial fracture in the UUO model [79] (Fig. 3).

Mitochondrial UPR (mtUPR) also optimizes mitochondria-ER interactions. For example, CHOP induces apoptosis during ER stress by lowering the protein level of Bcl-2 and by transferring Bax from the cytosol to mitochondria. In mitochondria, CHOP induction acts as an amplifier and integrator of apoptosis and is thought to be an early event of mtUPR [80]. Notably, although ER and mtUPR induce CHOP upregulation, there appears to be an overlap between the UPRs, but virtually each of them activate the transcription of a different set of target genes [81]. Interestingly, Zhong et al. showed that inhibition of ER stress with 4-phenylbutyric acid (4-PBA) mitigates kidney damage and mitochondrial apoptosis due to nephrotoxicity of 3-monochloropropane diol (3-MCPD) [82]. Another study showed that nicotine (NIC), a toxic component of smoke, can accelerate the progression of pre-existing kidney damage by disrupting mitochondrial structural integrity, triggering ER stress, and altering the expression of mitochondrial and ER stress genes [83]. Overall, these findings suggest that the crosstalk between ER and mitochondria has important implications in kidney disorders and that the regulation of both may be a potential therapeutic strategy for kidney protection.

### Endosome, Golgi, and ER crosstalk in kidney diseases

Endosomes are membrane-bound vesicles that transport various proteins between the Golgi and ER. Ashley et al. demonstrated that early and late endosome shrinkage and fission sites are spatially and temporally associated with ER contact sites [84]. Melissa et al. reported that endosomal fission requires transmembrane and coil domain family 1 (ER membrane protein, Transmembrane and Coiled-Coil Domain Family 1, TMCC1) and coronin 1C (endosomal localized actin regulator) at the ER-endosomal membrane contact site. TMCC1 functions to stabilize the ER membrane contact site (ER MCS) and has a cargo sorting domain on the endosomal bud. A previous study showed that TMCC1 depletion results in defects in cargo transport from late endosomes to the Golgi [85]. A crosstalk between ER and endosomes is also associated with kidney pathologies. Mucin 1 kidney disease (MKD) is an autosomal dominant hereditary tubular interstitial nephropathy characterized by progressive tubulointerstitial cyst formation. MKD is caused by a code shift mutation in mucin 1 (*MUC1*). Intracellular accumulation of misfolded proteins causes toxic proteinopathies, diseases without targeted therapies. Moran et al. showed that MKD is a toxic proteinopathy. The abnormal MUC1 protein (MUC1-fs) is trapped in endosomes containing transmembrane P24 trafficking protein 9 (TMED9) cargo receptors between the ER and the Golgi apparatus, preventing unfolded proteins from being transported to lysosomes through secretory pathways for degradation. As a result of the accumulation of MUC1-fs in tubular cells, the ATF6 branch that activates the UPR pathway eventually causes tubular damage (Fig. 3). BRD4780, as a candidate compound, has been shown to combine with TMED9 to release MUC1-fs and reroutes it for lysosomal degradation. BRD4780 is expected to become a leading compound for the treatment of renal toxic protein diseases [65, 86]. In addition, endosome and Golgi-associated degradation (EGAD) pathways have been identified to play important roles in ER clearance to prevent protein accumulation in the ER. Degradable substrates of the EGAD pathway include ER-resident membrane proteins (orosomucoid 2, Orm2), negative regulators of sphingolipid biosynthesis) required for lipid biosynthesis. Although this selective mechanism is found in yeast, Oliver et al. argued that it is not impossible to extract ubiquitinated ORM1-like (ORMDL) proteins from membranes in a process similar to EGAD. Given that elevated levels of the ORMDL3 protein are associated with diabetes, ulcerative colitis, Crohn’s disease, and asthma, the chelation of ORM family proteins from ER and its subsequent ubiquitin-dependent degradation may have pathophysiological implications [87].

## ER crosstalk with death mechanisms in kidney diseases

When continuous UPR fails to restore ER homeostasis, the downstream apoptosis pathways initiated by UPR, including IRE1-tumor necrosis factor (TNF) receptor-associated factor 2 (TRAF2)-apoptosis signal-regulating kinase 1 (ASK1)-JNK, Bax-Bak/IRE1, TRAF2-caspase-12/caspase-4, and PERK/ATG6/IRE1-CHOP, may eventually cause cell death [88, 89]. In addition to activating the apoptotic pathway described above to induce cell death, UPR is associated with pyroptosis, programmed necrosis, and ferroptosis. Previous studies have found that ER stress plays an important role in kidney pathologies caused by pyroptosis, and CHOP-caspase-11 triggered by overactivated ER stress may be an important pathway for pyroptosis-mediated IRI or hypoxic reoxygenation injury (HRI). Pretreatment with a low-dose of ER stress inducer tunicamycin can reduce IRI-induced pyroptosis and renal tissue damage. Moreover, silencing CHOP has been shown to decrease caspase-11 activity and IL-1β production, and it reduces IRI-induced pyroptosis of renal tubular epithelial cells [90, 91]. In addition, the occurrence of ferroptosis is also accompanied by the generation of ER stress. ER stress responses, especially the PERK-ATF4 pathway, often act as a protective mechanism to negatively regulate ferroptosis, especially in cancer cells involved in the formation of drug resistance [92]. However, some studies have shown that ER stress response promotes ferroptosis in some disease conditions. For example, considerable iron deposition, lipid radical accumulation, mitochondrial shrinkage, and other ferroptosis features have been observed in colonic mucosal cells in patients with ulcerative colitis (UC) and mouse UC models. The ER stress marker molecule GRP78 and PERK-ATF4-CHOP pathway are substantially activated in colonic epithelial cells of UC mice. Treatment with GSK414, an inhibitor of PERK, inhibits ferroptosis caused by dextran sulfate sodium salt (DSS), with a considerable decrease in the iron level and lipid peroxidation in colonic epithelial cells in mice [93]. Park et al. found that cigarette smoke condensate (WCSC) treatment induced ER stress, PERK, IRE1α, and ferroptosis pathways. Moreover, gene chip analysis showed that ER stress promotes the occurrence of ferroptosis. Under pathological conditions, the activation of ER stress pathway can exacerbate the occurrence of ferroptosis, confirming that ER stress can also cause ferroptosis under persistent or harsh disease conditions [94]. Recently, Zhao et al. demonstrated that the PERK-eIF2α-ATF4-CHOP pathway can inhibit ER stress and reduce cadmium-induced ferroptosis in cadmium-induced models of heavy metal toxic kidney injury. It was observed that ferroptosis and cadmium-induced nephrotoxicity were regulated by the MitoROS-ER stress-ferritinophagy axis [95] (Fig. 3).

## Treatment of kidney diseases by modulating ER

Studies have shown that ERdj3 and mesencephalic astrocyte-derived neurotrophic factor (MANF) that lack the KDEL motif can be used as indicators of glomerular ER stress [96]. ER stress marker levels in patients with AKI, such as RTN1A, are positively correlated with the severity of AKI [25, 97]. Cysteine-rich epidermal growth factor (EGF)-like domain 2 (CRELD2), a sensitive urine biomarker used to detect ER stress in several kidney disorders, including ischemic AKI. For example, the CRELD2 level is substantially increased in the urine of pediatric patients undergoing cardiac surgery within 6 h after surgery and patients with severe postoperative AKI [30, 98]. Circulating GRP78 and CHOP levels may be novel biomarkers for identifying diabetic kidney disease (DKD) [99]. Overall, these biomarkers may be beneficial for the early diagnosis, risk stratification, and monitoring of treatment response in patients with kidney diseases (Table 1).

**Table 1:** Summarize the biomarkers of ER stress associated with kidney disease.

Protein	Disease model	Function/dependency	Result	References
ANG	CKD, AKI (KTR)	Reflect the severity of the renal injury	Yes	Tavernier et al. [121]
CRELD2	AKI (TM, I/R) NS, ADTKD-UMOD	Urine ER stress biomarker, used for early diagnosis and guidance of ER-targeted therapy	Yes	Kim et al. [98]
ERdj3	PHN, PAN	Reflect glomerular ER stress	Yes	Tousson-Abouelazm et al. [96]
MANF	PHN, PAN, NS, AKI (TM, I/R)	Reflects ER stress in glomerulus and tubules	Yes	Tousson-Abouelazm et al. [96]; Kim et al. [122]
RTN1A	FAN, AAN, HIVAN, CKD (UUO, DN)	Mediator of UPR in kidney disease	Yes	Fan et al. [48]; Fan et al. [97]
GRP78 CHOP	T2DM	Biological indicators to distinguish DKD	Uncertain	Ma et al. [99]

*ANG* angiogenin, *CKD* chronic kidney disease, *AKI* acute kidney injury, *KTR* transplant failure, *CRELD2* cysteine rich with EGF like domains 2, *TM* tunicamycin, *I/R* ischemia–reperfusion, *NS* nephrotic syndrome, *ADTKD* autosomal dominant tubulointerstitial kidney disease, *UMOD* uromodulin, *PHN* passive Heymann nephritis, *PAN* puromycin aminonucleoside nephrosis, *MANF* mesencephalic astrocyte-derived neurotrophic factor, *RTN1A* Reticulon-1A, *FAN* folic acid nephropathy, *AAN* aristolochic acid nephropathy, *HIVAN* HIV-associated nephropathy, *UUO* unilateral ureteral obstruction, *DN* diabetic nephropathy, *GRP78* glucose-regulated protein 78, *T2DM* diabetes mellitus type 2, *DKD* diabetic kidney disease, *CHOP* C/EBP homologous protein.

Previous studies have shown that multiple molecules/drugs affect the outcome of kidney illnesses by modulating the UPR. First, the regulation of ER stress in kidney diseases could be achieved by targeting the IRE1-XBP1 and PERK-eIF2α axis. Angiopoietin (ANG) is a ribonuclease that has been shown to play a physiologically relevant ER-stress-mediated adaptive role in the translation control of kidney injury in an IRE1-XBP1-dependent manner [100]. Quercetin is one of the most available antioxidant flavonoids in the human diet and has been shown to inhibit the IRE1-TRAF2-JNK pathway in UUO [101], DN [102], asymmetric dimethylarginine (ADMA) [103], and cadmium-induced kidney injury [104], and maybe a powerful treatment option for targeting UPR. Salubrinal, a selective inhibitor of GADD34-phosphatase-1 (PP1) prevents dephosphorylation of eIF2α and protects cells from ER stress-induced apoptosis and hyperglycemia-induced podocyte damage [105], as well as kidney damage caused by toxic drugs, such as arsenic, paraquat, cyclosporine, cisplatin, and cadmium [106–109]. Chrysin (5,7 dihydroxyflavone) is a natural flavonoid found in propolis and mushrooms; it blocks hyperglycemic/diabetes-mediated ER stress/UPR and podocyte apoptosis by inhibiting PERK-EIF2α-ATF4-CHOP activation [22, 110]. CHOP expression is involved in several diseases, and CHOP deficiency can reduce renal fibrosis and inflammation [111]. Second, ER protein homeostasis could be regulated by inducing UPR. ER stress has been shown to elicit protective effects in several studies and pretreatment with tunicamycin protects mice from acute ischemic injury [112]. In a rat model of glomerulonephritis, tunicamycin significantly reduced mesangial proliferation and adhesion of Bowman capsules to glomerular clusters and proteinuria [113]. Overexpression of sNogo-B (N-terminal fragment of ER protein Nogo B) in circulation improved diabetic nephropathy by reducing proteinuria, ultrafiltration, and abnormal angiogenesis and protecting glomeruli [114]. The third type of agents is the AMPK activators. Metformin (an AMPK activator) inhibits ROS by inducing thioredoxin, an endogenous antioxidant molecule, and inhibition of GRP78 expression in an albumin-overloaded rat model protected tubular cells from albumin-loading induced ER stress [115]. In addition, metformin also inhibited ER stress and fibrosis in tunicamycin-induced AKI and UUO mouse models [116]. The fourth type is the chemical chaperones. Tauroursodeoxycholic acid (TUDCA) and 4-PBA could be used to treat kidney disorders. 4-PBA has been approved by the U.S. Food and Drug Administration (FDA) for use in children with urea cycle disorder [16]. Both compounds contribute to the alleviation of ER stress-related conditions, including renal fibrosis and DN [44, 117]. In STZ-induced DN, 4-PBA and TUDCA alleviate albuminuria and reduce the expression of GRP78, ATF6, PERK, JNK, and CHOP, as well as inflammatory mediators [118]. In addition, 4-PBA pretreatment reduces the expression of NLRP3 and inflammosomes [119]. The findings indicate that 4-PBA and TUDCA are potential drugs to reduce renal fibrosis. However, the multiple biological effects of drugs could lead to off-target effects. Therefore, the development of drugs with precise ER targeting has become an important research focus in the prevention and treatment of related diseases.

Currently, research on ER-targeted drugs is still in its infancy, and knowledge of the types of active targeting molecules and their quantities is limited. Moreover, it would be difficult to meet the needs of developing a multifunctional ER-targeted nanomedicine using these targeted molecules. However, the clustered regularly interspaced short palindromic repeats (CRISPR)/Cas9 genome editing technology could be used to comprehensively test the efficiencies of these drugs under different ER stress scenarios. Human-induced pluripotent stem cells (hiPSCs) could be established for disease modeling, mechanistic research, and future drug discovery [30]. Overall, it is believed that these novel technologies would greatly facilitate the implementation of precision medicine in ER stress-mediated kidney pathologies and may lead to the development of highly targeted ER stress modulators for individual mutations. Notably, epigenetic enzyme block is also a promising target for improving kidney damage. Recently, epigenetic kinetics mediated by H3K9 and H3K27 histone methylation has been key to regulating ATF4 and XBP1 transcription factor expression, providing potential treatment strategies for regulating the pathological consequences of acute ER stress responses. Pharmacological inhibition of histone demethylases (HDMs) (KDM4C and Jumonji domain-containing protein-3) is considered to help eliminate pathological consequences triggered by maladaptive UPR activation during kidney injury [120] (Table 2).

**Table 2:** Summarized the therapeutic effect of modulators targeting ER homeostasis in renal diseases.

Chemical	Mechanism of action	Animal model	Therapeutic effect	References
Quercetin	IRE1 Rnase activatior	UUO, STZ-DN, Cadmium ADMA	ROS↓ MCP-1↓ TGF-β↓ Apoptosis↓	Jones et al. [101]; Anjaneyulu et al. [102]; Morales et al. [104]; Guo et al. [103]
Salubrinal	eIF2α phosphatase inhibitor	Cyclospo-rine Cadmium Cisplatin	ER Stress↓ Apoptosis↓ epithelial phenotypic changes (EPCs)↓ ROS↑ Apoptosis↑	Pallet et al. [107]; Komoike et.al. [108]; Wu et al. [109]
Chrysin	eIF2α phosphatase inhibitor	db/db-DN	ER Stress↓ Slit -diaphragm protein ↓ Apoptosis↓	Kang et al. [110]
Tunicamycin	Modulation of ER proteins	IR anti-Thy1 nephritis	Kidney injury↓ GRP78↑	Prachasilchai et al. [112]; Inagi et al. [113]
sNogo-B	Modulation of ER proteins	STZ-DN	Urinary albumin↓ Filtration↓ VEGF-A↑ Proliferation of GECs↓ ER Stress↓	Hernandez-Diaz et al. [114]
Metformin	AMPK activation	UUO AKI(TM) Protein-Overload proteinuria rats	Fibrosis↓ Apoptosis↓	Kim et al. [116]; Lee et al. [115]
4-PBA TUDCA	Chemical Chaperones	uIR, UUO	Fibrosis↓ Apoptosis↓ Inflammation↓ Autophagy↓	Liu et al. [117] Shu et al. [44]
KDM4C JMJD3	Histone methylation regulates the expression of ATF4 and XBP-1	AKI(TG) UUO	ER stress during kidney injury↓	Diaz-Bulnes et al. [120]

*UUO* unilateral ureteral obstruction, *STZ-DN* streptozotocin-induced diabetic nephropathy, *ADMA* asymmetric dimethylarginine, *ROS* reactive oxygen species, *MCP-1* monocyte chemoattractant protein-1, *TGF-β* transforming growth factor-β, *TM* tunicamycin, *I/R* ischemia–reperfusion, *GRP78* glucose-regulated protein 78, *VEGF-A* vascular endothelial growth factor-A, *GECs* glomerular endothelial cells, *TM* tunicamycin, *TG* thapsigargin, *TUDCA* tauroursodeoxycholic acid, *4-PBA* 4-phenylbutyric acid.

“↑/↓” in Therapeutic effect represents an increase or decrease compared to controls.

## Conclusions

In summary, dysregulation of ER homeostasis in renal resident cells not only affects characteristic pathophysiological markers but also triggers the onset and progression of various kidney diseases by promoting renal inflammation and fibrosis. Restoration of ER homeostasis through UPR, ERAD, and autophagy presents a potential for the treatment of kidney diseases. As ER regulators may also have a variety of biological effects and off-target effects, further studies are necessary to confirm the specificity and safety of targeted agents.

## Data Availability

There are no experimental datasets, given that this is a review article that is prepared based on a literature review.

## References

[1] Chen S, Novick P, Ferro-Novick S (2013). ER structure and function. Curr Opin Cell Biol.

[2] Westrate LM, Lee JE, Prinz WA, Voeltz GK (2015). Form follows function: the importance of endoplasmic reticulum shape. Annu Rev Biochem.

[3] Csordás G, Weaver D, Hajnóczky G (2018). Endoplasmic reticulum-mitochondrial contactology: structure and signaling functions. Trends Cell Biol.

[4] Saraste J (2016). Spatial and functional aspects of ER-Golgi Rabs and tethers. Front Cell Dev Biol.

[5] Chino H, Mizushima N (2020). ER-Phagy: quality control and turnover of endoplasmic reticulum. Trends Cell Biol.

[6] Ni L, Yuan C, Wu X (2021). Endoplasmic reticulum stress in diabetic nephrology: regulation, pathological role, and therapeutic potential. Oxid Med Cell Longev.

[7] Moon HW, Han HG, Jeon YJ (2018). Protein quality control in the endoplasmic reticulum and cancer. Int J Mol Sci.

[8] Hwang J, Qi L (2018). Quality control in the endoplasmic reticulum: crosstalk between ERAD and UPR pathways. Trends Biochem Sci.

[9] Zito E (2019). Targeting ER stress/ER stress response in myopathies. Redox Biol.

[10] Smith M, Wilkinson S (2017). ER homeostasis and autophagy. Essays Biochem.

[11] Nakada EM, Sun R, Fujii U, Martin JG (2021). The impact of endoplasmic reticulum-associated protein modifications, folding and degradation on lung structure and function. Front Physiol.

[12] Jiang Y, Tao Z, Chen H, Xia S (2021). Endoplasmic reticulum quality control in immune cells. Front Cell Dev Biol.

[13] Yamamoto K, Sato T, Matsui T, Sato M, Okada T, Yoshida H (2007). Transcriptional induction of mammalian ER quality control proteins is mediated by single or combined action of ATF6alpha and XBP1. Dev Cell.

[14] Jin JK, Blackwood EA, Azizi K, Thuerauf DJ, Fahem AG, Hofmann C (2017). ATF6 decreases myocardial ischemia/reperfusion damage and links ER stress and oxidative stress signaling pathways in the heart. Circ Res.

[15] Szegezdi E, Logue SE, Gorman AM, Samali A (2006). Mediators of endoplasmic reticulum stress-induced apoptosis. EMBO Rep.

[16] Chen JH, Wu CH, Chiang CK (2021). Therapeutic approaches targeting proteostasis in kidney disease and fibrosis. Int J Mol Sci.

[17] Fregno I, Molinari M (2019). Proteasomal and lysosomal clearance of faulty secretory proteins: ER-associated degradation (ERAD) and ER-to-lysosome-associated degradation (ERLAD) pathways. Crit Rev Biochem Mol Biol.

[18] Margariti A, Li H, Chen T, Martin D, Vizcay-Barrena G, Alam S (2013). XBP1 mRNA splicing triggers an autophagic response in endothelial cells through BECLIN-1 transcriptional activation. J Biol Chem.

[19] Li W, He P, Huang Y, Li YF, Lu J, Li M (2021). Selective autophagy of intracellular organelles: recent research advances. Theranostics.

[20] Murton M, Goff-Leggett D, Bobrowska A, Garcia Sanchez JJ, James G, Wittbrodt E (2021). Burden of chronic kidney disease by KDIGO categories of glomerular filtration rate and albuminuria: a systematic review. Adv Ther.

[21] Garzon FT, Simanowski UA, Berger MR, Schmähl D, Kommerell B, Seitz HK (1987). Acetoxymethyl-methylnitrosamine (AMMN) induced colorectal carcinogenesis is stimulated by chronic alcohol consumption. Alcohol Alcohol Suppl.

[22] Ricciardi CA, Gnudi L (2019). Endoplasmic reticulum stress in chronic kidney disease. New molecular targets from bench to the bedside. G Ital Nefrol.

[23] Kalantar-Zadeh K, Jafar TH, Nitsch D, Neuen BL, Perkovic V (2021). Chronic kidney disease. Lancet.

[24] Cybulsky AV (2017). Endoplasmic reticulum stress, the unfolded protein response and autophagy in kidney diseases. Nat Rev Nephrol.

[25] Gallazzini M, Pallet N (2018). Endoplasmic reticulum stress and kidney dysfunction. Biol Cell.

[26] Ohse T, Inagi R, Tanaka T, Ota T, Miyata T, Kojima I (2006). Albumin induces endoplasmic reticulum stress and apoptosis in renal proximal tubular cells. Kidney Int.

[27] Mohammed-Ali Z, Lu C, Marway MK, Carlisle RE, Ask K, Lukic D (2017). Endoplasmic reticulum stress inhibition attenuates hypertensive chronic kidney disease through reduction in proteinuria. Sci Rep.

[28] Nademi S, Dickhout JG (2019). Protein misfolding in endoplasmic reticulum stress with applications to renal diseases. Adv Protein Chem Struct Biol.

[29] Pieri M, Stefanou C, Zaravinos A, Erguler K, Stylianou K, Lapathitis G (2014). Evidence for activation of the unfolded protein response in collagen IV nephropathies. J Am Soc Nephrol.

[30] Park SJ, Kim Y, Chen YM (2019). Endoplasmic reticulum stress and monogenic kidney diseases in precision nephrology. Pediatr Nephrol.

[31] Ricciardi CA, Gnudi L (2020). The endoplasmic reticulum stress and the unfolded protein response in kidney disease: Implications for vascular growth factors. J Cell Mol Med.

[32] Navarro-Betancourt JR, Papillon J, Guillemette J, Iwawaki T, Chung CF, Cybulsky AV (2020). Role of IRE1α in podocyte proteostasis and mitochondrial health. Cell Death Discov.

[33] Hassan H, Tian X, Inoue K, Chai N, Liu C, Soda K (2016). Essential role of X-box binding protein-1 during endoplasmic reticulum stress in podocytes. J Am Soc Nephrol.

[34] Ishikawa Y, Fedeles S, Marlier A, Zhang C, Gallagher AR, Lee AH (2019). Spliced XBP1 rescues renal interstitial inflammation due to loss of Sec63 in collecting ducts. J Am Soc Nephrol.

[35] Ferrè S, Deng Y, Huen SC, Lu CY, Scherer PE, Igarashi P (2019). Renal tubular cell spliced X-box binding protein 1 (Xbp1s) has a unique role in sepsis-induced acute kidney injury and inflammation. Kidney Int.

[36] Fedeles SV, So JS, Shrikhande A, Lee SH, Gallagher AR, Barkauskas CE (2015). Sec63 and Xbp1 regulate IRE1α activity and polycystic disease severity. J Clin Investig.

[37] Li Y, Jiang Y, Zhou W, Wu Y, Zhang S, Ding G (2022). Maintaining homeostasis of mitochondria and endoplasmic reticulum with NSC228155 alleviates cisplatin-induced acute kidney injury. Free Radic Biol Med.

[38] Peyrou M, Cribb AE (2007). Effect of endoplasmic reticulum stress preconditioning on cytotoxicity of clinically relevant nephrotoxins in renal cell lines. Toxicol Vitr.

[39] Chandrika BB, Yang C, Ou Y, Feng X, Muhoza D, Holmes AF (2015). Endoplasmic reticulum stress-induced autophagy provides cytoprotection from chemical hypoxia and oxidant injury and ameliorates renal ischemia-reperfusion injury. PLoS ONE.

[40] Wang Y, Tian J, Qiao X, Su X, Mi Y, Zhang R (2015). Intermedin protects against renal ischemia-reperfusion injury by inhibiting endoplasmic reticulum stress. BMC Nephrol.

[41] Shan B, Wang X, Wu Y, Xu C, Xia Z, Dai J (2017). The metabolic ER stress sensor IRE1α suppresses alternative activation of macrophages and impairs energy expenditure in obesity. Nat Immunol.

[42] Shu S, Wang H, Zhu J, Liu Z, Yang D, Wu W (2021). Reciprocal regulation between ER stress and autophagy in renal tubular fibrosis and apoptosis. Cell Death Dis.

[43] El Karoui K, Viau A, Dellis O, Bagattin A, Nguyen C, Baron W (2016). Endoplasmic reticulum stress drives proteinuria-induced kidney lesions via Lipocalin 2. Nat Commun.

[44] Shu S, Zhu J, Liu Z, Tang C, Cai J, Dong Z (2018). Endoplasmic reticulum stress is activated in post-ischemic kidneys to promote chronic kidney disease. EBioMedicine.

[45] Jao TM, Nangaku M, Wu CH, Sugahara M, Saito H, Maekawa H (2019). ATF6α downregulation of PPARα promotes lipotoxicity-induced tubulointerstitial fibrosis. Kidney Int.

[46] Pajares M, Cuadrado A, Rojo AI (2017). Modulation of proteostasis by transcription factor NRF2 and impact in neurodegenerative diseases. Redox Biol.

[47] Cullinan SB, Zhang D, Hannink M, Arvisais E, Kaufman RJ, Diehl JA (2003). Nrf2 is a direct PERK substrate and effector of PERK-dependent cell survival. Mol Cell Biol.

[48] Fan Y, Xiao W, Li Z, Li X, Chuang PY, Jim B (2015). RTN1 mediates progression of kidney disease by inducing ER stress. Nat Commun.

[49] Yan M, Shu S, Guo C, Tang C, Dong Z (2018). Endoplasmic reticulum stress in ischemic and nephrotoxic acute kidney injury. Ann Med.

[50] Binet F, Sapieha P (2015). ER stress and angiogenesis. Cell Metab.

[51] Wu Y, Li Y, Jiang T, Yuan Y, Li R, Xu Z (2018). Reduction of cellular stress is essential for Fibroblast growth factor 1 treatment for diabetic nephropathy. J Cell Mol Med.

[52] Tan X, Yu L, Yang R, Tao Q, Xiang L, Xiao J (2020). Fibroblast growth factor 10 attenuates renal damage by regulating endoplasmic reticulum stress after ischemia-reperfusion injury. Front Pharm.

[53] Zhang MZ, Wang Y, Paueksakon P, Harris RC (2014). Epidermal growth factor receptor inhibition slows progression of diabetic nephropathy in association with a decrease in endoplasmic reticulum stress and an increase in autophagy. Diabetes.

[54] Anusornvongchai T, Nangaku M, Jao TM, Wu CH, Ishimoto Y, Maekawa H (2018). Palmitate deranges erythropoietin production via transcription factor ATF4 activation of unfolded protein response. Kidney Int.

[55] Høyer-Hansen M, Jäättelä M (2007). Connecting endoplasmic reticulum stress to autophagy by unfolded protein response and calcium. Cell Death Differ.

[56] Yuan S, Liang X, He W, Liang M, Jin J, He Q (2021). ATF4-dependent heme-oxygenase-1 attenuates diabetic nephropathy by inducing autophagy and inhibiting apoptosis in podocyte. Ren Fail.

[57] Wang L, Pan Y, Yang F, Guo X, Peng J, Wang X (2022). New sight into interaction between endoplasmic reticulum stress and autophagy induced by vanadium in duck renal tubule epithelial cells. Chem Biol Interact.

[58] Kawakami T, Inagi R, Takano H, Sato S, Ingelfinger JR, Fujita T (2009). Endoplasmic reticulum stress induces autophagy in renal proximal tubular cells. Nephrol Dial Transpl.

[59] Dong G, Liu Y, Zhang L, Huang S, Ding HF, Dong Z (2015). mTOR contributes to ER stress and associated apoptosis in renal tubular cells. Am J Physiol Ren Physiol.

[60] Jiang S, Lin Y, Yao H, Yang C, Zhang L, Luo B (2018). The role of unfolded protein response and ER-phagy in quantum dots-induced nephrotoxicity: an in vitro and in vivo study. Arch Toxicol.

[61] Li H, Qu L, Zhou R, Wu Y, Zhou S, Zhang Y (2020). TRIM13 inhibits cell migration and invasion in clear-cell renal cell carcinoma. Nutr Cancer.

[62] Li Y, Ren D, Shen Y, Zheng X, Xu G (2020). Altered DNA methylation of TRIM13 in diabetic nephropathy suppresses mesangial collagen synthesis by promoting ubiquitination of CHOP. EBioMedicine.

[63] Dorotea D, Jiang S, Pak ES, Son JB, Choi HG, Ahn SM (2022). Pan-Src kinase inhibitor treatment attenuates diabetic kidney injury via inhibition of Fyn kinase-mediated endoplasmic reticulum stress. Exp Mol Med.

[64] Inoue T, Maekawa H, Inagi R (2019). Organelle crosstalk in the kidney. Kidney Int.

[65] Hasegawa S, Inagi R (2020). Organelle stress and crosstalk in kidney disease. Kidney360.

[66] Han JM, Periwal V (2019). A mathematical model of calcium dynamics: obesity and mitochondria-associated ER membranes. PLoS Comput Biol.

[67] van Vliet AR, Verfaillie T, Agostinis P (2014). New functions of mitochondria associated membranes in cellular signaling. Biochim Biophys Acta.

[68] Gao P, Yang W, Sun L (2020). Mitochondria-associated endoplasmic reticulum membranes (MAMs) and their prospective roles in kidney disease. Oxid Med Cell Longev.

[69] Igwebuike C, Yaglom J, Huiting L, Feng H, Campbell JD, Wang Z (2020). Cross organelle stress response disruption promotes gentamicin-induced proteotoxicity. Cell Death Dis.

[70] Yang M, Zhao L, Gao P, Zhu X, Han Y, Chen X (2019). DsbA-L ameliorates high glucose induced tubular damage through maintaining MAM integrity. EBioMedicine.

[71] Yang M, Han Y, Luo S, Xiong X, Zhu X, Zhao H (2021). MAMs protect against ectopic fat deposition and lipid-related kidney damage in DN patients. Front Endocrinol.

[72] Li C, Li L, Yang M, Yang J, Zhao C, Han Y (2022). PACS-2 ameliorates tubular injury by facilitating endoplasmic reticulum-mitochondria contact and mitophagy in diabetic nephropathy. Diabetes.

[73] Xue M, Fang T, Sun H, Cheng Y, Li T, Xu C (2021). PACS-2 attenuates diabetic kidney disease via the enhancement of mitochondria-associated endoplasmic reticulum membrane formation. Cell Death Dis.

[74] Cao Y, Chen Z, Hu J, Feng J, Zhu Z, Fan Y (2021). Mfn2 regulates high glucose-induced MAMs dysfunction and apoptosis in podocytes via PERK pathway. Front Cell Dev Biol.

[75] Wang X, Cao H, Fang Y, Bai H, Chen J, Xing C (2022). Activation of endoplasmic reticulum-mitochondria coupling drives copper-induced autophagy in duck renal tubular epithelial cells. Ecotoxicol Environ Saf.

[76] Xie Y, Jing E, Cai H, Zhong F, Xiao W, Gordon RE (2022). Reticulon-1A mediates diabetic kidney disease progression through endoplasmic reticulum-mitochondrial contacts in tubular epithelial cells. Kidney Int.

[77] Peng J, Peng C, Wang L, Cao H, Xing C, Li G (2022). Endoplasmic reticulum-mitochondria coupling attenuates vanadium-induced apoptosis via IP(3)R in duck renal tubular epithelial cells. J Inorg Biochem.

[78] Inagi R (2022). Organelle stress and metabolic derangement in kidney disease. Int J Mol Sci.

[79] Noh MR, Woo CH, Park MJ, In Kim J, Park KM (2018). Ablation of C/EBP homologous protein attenuates renal fibrosis after ureteral obstruction by reducing autophagy and microtubule disruption. Biochim Biophys Acta Mol Basis Dis.

[80] Martínez-Klimova E, Aparicio-Trejo OE, Gómez-Sierra T, Jiménez-Uribe AP, Bellido B, Pedraza-Chaverri J (2020). Mitochondrial dysfunction and endoplasmic reticulum stress in the promotion of fibrosis in obstructive nephropathy induced by unilateral ureteral obstruction. Biofactors.

[81] Horibe T, Hoogenraad NJ (2007). The chop gene contains an element for the positive regulation of the mitochondrial unfolded protein response. PLoS ONE.

[82] Zhong Y, Jin C, Han J, Zhu J, Liu Q, Sun D (2021). Inhibition of ER stress attenuates kidney injury and apoptosis induced by 3-MCPD via regulating mitochondrial fission/fusion and Ca(2+) homeostasis. Cell Biol Toxicol.

[83] Jiang YJ, Cui S, Luo K, Ding J, Nan QY, Piao SG (2021). Nicotine exacerbates tacrolimus-induced renal injury by programmed cell death. Korean J Intern Med.

[84] Rowland AA, Chitwood PJ, Phillips MJ, Voeltz GK (2014). ER contact sites define the position and timing of endosome fission. Cell.

[85] Hoyer MJ, Chitwood PJ, Ebmeier CC, Striepen JF, Qi RZ, Old WM (2018). A novel class of ER membrane proteins regulates ER-associated endosome fission. Cell.

[86] Dvela-Levitt M, Kost-Alimova M, Emani M, Kohnert E, Thompson R, Sidhom EH (2019). Small molecule targets TMED9 and promotes lysosomal degradation to reverse proteinopathy. Cell.

[87] Schmidt O, Weyer Y, Baumann V, Widerin MA, Eising S, Angelova M (2019). Endosome and Golgi-associated degradation (EGAD) of membrane proteins regulates sphingolipid metabolism. EMBO J.

[88] Avivar-Valderas A, Salas E, Bobrovnikova-Marjon E, Diehl JA, Nagi C, Debnath J (2011). PERK integrates autophagy and oxidative stress responses to promote survival during extracellular matrix detachment. Mol Cell Biol.

[89] Xue LX, Liu HY, Cui Y, Dong Y, Wang JQ, Ji QY (2017). Neuroprotective effects of Activin A on endoplasmic reticulum stress-mediated apoptotic and autophagic PC12 cell death. Neural Regen Res.

[90] Yang JR, Yao FH, Zhang JG, Ji ZY, Li KL, Zhan J (2014). Ischemia-reperfusion induces renal tubule pyroptosis via the CHOP-caspase-11 pathway. Am J Physiol Ren Physiol.

[91] Li N, Wang Y, Wang X, Sun N, Gong YH (2022). Pathway network of pyroptosis and its potential inhibitors in acute kidney injury. Pharm Res.

[92] Lee YS, Lee DH, Choudry HA, Bartlett DL, Lee YJ (2018). Ferroptosis-induced endoplasmic reticulum stress: cross-talk between ferroptosis and apoptosis. Mol Cancer Res.

[93] Xu M, Tao J, Yang Y, Tan S, Liu H, Jiang J (2020). Ferroptosis involves in intestinal epithelial cell death in ulcerative colitis. Cell Death Dis.

[94] Park EJ, Park YJ, Lee SJ, Lee K, Yoon C (2019). Whole cigarette smoke condensates induce ferroptosis in human bronchial epithelial cells. Toxicol Lett.

[95] Zhao C, Yu D, He Z, Bao L, Feng L, Chen L (2021). Endoplasmic reticulum stress-mediated autophagy activation is involved in cadmium-induced ferroptosis of renal tubular epithelial cells. Free Radic Biol Med.

[96] Tousson-Abouelazm N, Papillon J, Guillemette J, Cybulsky AV (2020). Urinary ERdj3 and mesencephalic astrocyte-derived neutrophic factor identify endoplasmic reticulum stress in glomerular disease. Lab Investig.

[97] Fan Y, Xiao W, Lee K, Salem F, Wen J, He L (2017). Inhibition of reticulon-1A-mediated endoplasmic reticulum stress in early AKI attenuates renal fibrosis development. J Am Soc Nephrol.

[98] Kim Y, Park SJ, Manson SR, Molina CA, Kidd K, Thiessen-Philbrook H (2017). Elevated urinary CRELD2 is associated with endoplasmic reticulum stress-mediated kidney disease. JCI Insight.

[99] Ma N, Xu N, Yin D, Zheng P, Liu W, Wang G (2021). Levels of circulating GRP78 and CHOP in endoplasmic reticulum stress pathways in Chinese type 2 diabetic kidney disease patients. Medicine.

[100] Mami I, Bouvier N, El Karoui K, Gallazzini M, Rabant M, Laurent-Puig P (2016). Angiogenin mediates cell-autonomous translational control under endoplasmic reticulum stress and attenuates kidney injury. J Am Soc Nephrol.

[101] Jones EA, Shahed A, Shoskes DA (2000). Modulation of apoptotic and inflammatory genes by bioflavonoids and angiotensin II inhibition in ureteral obstruction. Urology.

[102] Anjaneyulu M, Chopra K (2004). Quercetin, an anti-oxidant bioflavonoid, attenuates diabetic nephropathy in rats. Clin Exp Pharm Physiol.

[103] Guo W, Ding J, Zhang A, Dai W, Liu S, Diao Z (2014). The inhibitory effect of quercetin on asymmetric dimethylarginine-induced apoptosis is mediated by the endoplasmic reticulum stress pathway in glomerular endothelial cells. Int J Mol Sci.

[104] Morales AI, Vicente-Sánchez C, Sandoval JM, Egido J, Mayoral P, Arévalo MA (2006). Protective effect of quercetin on experimental chronic cadmium nephrotoxicity in rats is based on its antioxidant properties. Food Chem Toxicol.

[105] Fang L, Zhou Y, Cao H, Wen P, Jiang L, He W (2013). Autophagy attenuates diabetic glomerular damage through protection of hyperglycemia-induced podocyte injury. PLoS ONE.

[106] Matsuoka M, Komoike Y (2015). Experimental evidence shows salubrinal, an eIF2α dephosphorylation inhibitor, reduces xenotoxicant-induced cellular damage. Int J Mol Sci.

[107] Pallet N, Bouvier N, Bendjallabah A, Rabant M, Flinois JP, Hertig A (2008). Cyclosporine-induced endoplasmic reticulum stress triggers tubular phenotypic changes and death. Am J Transpl.

[108] Komoike Y, Inamura H, Matsuoka M (2012). Effects of salubrinal on cadmium-induced apoptosis in HK-2 human renal proximal tubular cells. Arch Toxicol.

[109] Wu CT, Sheu ML, Tsai KS, Chiang CK, Liu SH (2011). Salubrinal, an eIF2α dephosphorylation inhibitor, enhances cisplatin-induced oxidative stress and nephrotoxicity in a mouse model. Free Radic Biol Med.

[110] Kang MK, Park SH, Kim YH, Lee EJ, Antika LD, Kim DY (2017). Chrysin ameliorates podocyte injury and slit diaphragm protein loss via inhibition of the PERK-eIF2α-ATF-CHOP pathway in diabetic mice. Acta Pharm Sin.

[111] Chen BL, Sheu ML, Tsai KS, Lan KC, Guan SS, Wu CT (2015). CCAAT-enhancer-binding protein homologous protein deficiency attenuates oxidative stress and renal ischemia-reperfusion injury. Antioxid Redox Signal.

[112] Prachasilchai W, Sonoda H, Yokota-Ikeda N, Oshikawa S, Aikawa C, Uchida K (2008). A protective role of unfolded protein response in mouse ischemic acute kidney injury. Eur J Pharm.

[113] Inagi R, Kumagai T, Nishi H, Kawakami T, Miyata T, Fujita T (2008). Preconditioning with endoplasmic reticulum stress ameliorates mesangioproliferative glomerulonephritis. J Am Soc Nephrol.

[114] Hernandez-Diaz I, Pan J, Ricciardi CA, Bai X, Ke J, White KE (2019). Overexpression of circulating soluble Nogo-B improves diabetic kidney disease by protecting the vasculature. Diabetes.

[115] Lee EK, Jeong JU, Chang JW, Yang WS, Kim SB, Park SK (2012). Activation of AMP-activated protein kinase inhibits albumin-induced endoplasmic reticulum stress and apoptosis through inhibition of reactive oxygen species. Nephron Exp Nephrol.

[116] Kim H, Moon SY, Kim JS, Baek CH, Kim M, Min JY (2015). Activation of AMP-activated protein kinase inhibits ER stress and renal fibrosis. Am J Physiol Ren Physiol.

[117] Liu SH, Yang CC, Chan DC, Wu CT, Chen LP, Huang JW (2016). Chemical chaperon 4-phenylbutyrate protects against the endoplasmic reticulum stress-mediated renal fibrosis in vivo and in vitro. Oncotarget.

[118] Qi W, Mu J, Luo ZF, Zeng W, Guo YH, Pang Q (2011). Attenuation of diabetic nephropathy in diabetes rats induced by streptozotocin by regulating the endoplasmic reticulum stress inflammatory response. Metabolism.

[119] Wang J, Wen Y, Lv LL, Liu H, Tang RN, Ma KL (2015). Involvement of endoplasmic reticulum stress in angiotensin II-induced NLRP3 inflammasome activation in human renal proximal tubular cells in vitro. Acta Pharm Sin.

[120] Diaz-Bulnes P, Saiz ML, Corte-Iglesias V, Rodrigues-Diez RR, Bernardo Florez A, Ruiz (2022). Demethylation of H3K9 and H3K27 contributes to the tubular renal damage triggered by endoplasmic reticulum stress. Antioxidants.

[121] Tavernier Q, Mami I, Rabant M, Karras A, Laurent-Puig P, Chevet E (2017). Urinary angiogenin reflects the magnitude of kidney injury at the infrahistologic level. J Am Soc Nephrol.

[122] Kim Y, Lee H, Manson SR, Lindahl M, Evans B, Miner JH (2016). Mesencephalic astrocyte-derived neurotrophic factor as a urine biomarker for endoplasmic reticulum stress-related kidney diseases. J Am Soc Nephrol.

